# The Experiences of an Unsuccessful Practitioner

**Published:** 1875-09

**Authors:** 


### MISCELLANEOUS.

### The Experiences of an Unsuccessful Practitioner.

*To the Editor of the Medical Record:*

DEAR SIR:—Davids are always singing the attractions of the medical profession and never lack an audience; Jeremiahs utter their lamentations in vain, and are of no account.

Nevertheless I have reason to think that Jeremiahs are by far the more numerous, and have a good deal on their side. As one of the Jeremiahs, therefore, I trust I may be allowed my growl, if only for fair play. If what I write savors of egotism, I here once for all make an humble apology; but egotistical or not, my only wish is to set down the facts of my case, now that I take my leave of medicine, for others' benefit.

About eleven years ago I commenced the study of medicine, immediately on graduating from one of the best literary colleges in the country.

After three years spent in hard study and faithful attendance on lectures, I took my degree at Bellevue Hospital Medical College; then I passed the examination and commenced my term of service in the Hospital (Bellevue). On the expiration of my eighteen months I went abroad, and spent a year of hard work in Germany.

On my return I received the appointment to the surgical class of a large dispensary, and also opened an office in a thickly settled district near Washington Parade Ground.

I wrote several articles for the medical journals—indeed you published some of them—on which I was complimented by many of the older members of the profession. Then I joined different societies, and employed my ample leisure working at chemistry, and with the microscope, and in reading the journals. So you see, my education was much more extensive than that usually enjoyed by students.

I trust these details will be pardoned. I wish to show that the oft-repeated story of non-success from coquetting with your business, and allowing other concerns to take your time, was not the case in this instance.

This was all very well, and gave me great pleasure in the doing, for I was an enthusiast about medicine, and would have liked nothing better than to have pursued it for its own sake; but, unfortunately, the profession had been adopted by me as an ultimate dependence for necessary bread and butter. Now, my neat and elegant account books showed, at the end of the second year in city practice, just two entries, relating to two patients, from neither of whom had anything been collected.

I saw my capital dwindling, and resolved to make a move at once, as I could not afford to remain longer where I was.

My location was changed to a town beyond the immediate influences of the metropolis, nearly one hundred miles away from the city. It was a place of some four thousand inhabitants, many of whom were very well off, and as there were but three physicians there, I thought a fourth might find something to do.

Well, the first year I made $500, barely enough to cover expenses for living, a horse and wagon being indispensable in a place where often I had to ride many miles. The work was hard; that I had bargained for, but the bane of the place was its "beat" element. Had half of those who consulted me paid, and most of them were fully able to do so, I would have done a stunning business; as it was, my $400 just represented the payments of one-fifth of my patients, the remaining four-fifths being still my debtors.

The second year saw the $400 of the first sink to $100. The cause of this phenomenon is easily explained. One of the original, old-established doctors died, and as soon as the fact became known, a certain shrewd individual from New York made up his mind that he could not do a better thing than to come here and give himself out as the successor to the deceased doctor. Unfortunately for his calculations the same intention animated the minds of no lees a number than five equally shrewd individuals, and Beeler's Quarters was plethoric with doctors. These new comers at first set about to vindicate, each for himself, the title of being the only original Jacobs. But competition did not enliven trade, and at last things got so bad that each man of the six set up a private dispensary of his own, and quarreled with his neighbor over the few charity patients who ventured on availing themselves of the abundant provisions for medical relief. Finally poor people were induced to submit their ailments to one of the dispensary proprietors, who had the reputation of always prescribing tinctures, and as the opposition made use of fluid extracts the practice of the alcohol man grew apace. But the six soon tired of this business of competition and free dispensaries, medicine included, and then they banded themselves together into a mutual benefit and admiration society against an innocent though unappreciative community.

Since the spring floods from the medical colleges four newly-made graduates have added themselves to our number, they likewise having heard that there was a dead man's place to be filled, and supposing, in their ignorance, that they had found "an opening." I need not dilate on the effects of all this on my business. When I tell you that since the first of February, 1874, up to the present time, I have pocketed the magnificent sum of $50 from my practice, I have reason to believe that I am ahead of some of my fellow unfortunates. This is not all. There have lately come here a rubbing doctor and his wife, who at once set every sick and imaginary sick person mad on the subject of their peculiar practice. Not only we, late comers, but the old and well-established physicians of the place have been made to suffer by the seductive pamphlet's influence. It is only the ignorant that patronize these people, who are a pair of as pestilent, vulgar, and ill-educated quacks as it has ever been my ill-fortune to meet, but the most prominent and intelligent minister of the place has sent his wife to be rubbed, and the valetudinarians of all the churches followed the lead.

When the man of greatest importance in the town is gained over by a discourse on the certain efficacy of expelling disease, as an entity, by rubbing and stimulating "the pores," what chance can a modest professional sign have as attraction, when placed in competition with a lively colored representation of a joyful ex-cripple throwing away his crutches at the touch of a long-haired individual near by.

To-day I say good-bye to the profession. It is greatly to my regret to be obliged to do it. Willingly would I wait the ten years which the gentlemen under whom I served at the hospital tell me will bring success, for my desire is, above all things, to remain in the ranks. But stern necessity knows no law of choice.

Nearly eleven years ago I commenced with a capital of eight thousand dollars, which I expected would pay for education and give me something to live on till my practice should be sufficient to bring me a living. The facts show that I have nearly consumed my capital, and am farther than ever from the chance of making a living by medicine. I shall take the thousand or so I have left and try to get in some mercantile business, which, no matter what it may be, can hardly promise worse than the certain starvation if medicine be longer relied on. Fortunately I have only myself to consider; if I fail in a new undertaking no one will be dragged down with me. What is the secret of my non-success? It is not repulsiveness of person; it is not lack of decent manners; it is not extreme youth; it is not excessive age; it is not inattention or want of earnestness in such business as I have had; it is not the commission of some unfortunate mistake which has shaken people's confidence; it is not lack of proper technical education, yet even the hod-carrier makes more money than I, and I pay the girl in my kitchen a stipend far beyond the amount of my own receipts.

I recollect hearing a genial and popular professor once, giving his closing lecture to his class, say: "Gentlemen, a great many of you, over a hundred and a score, will be sent out from this school this year—many more from all the schools over the land—and besides, there is an absolute increase in the numberof physicians; but we want all of you; this great and glorious country cannot have too many."

Then came something about "our glorious, our noble profession," which was applauded to the echo.

Now, I respectfully submit this is all wrong. Platitudes are very well, but when young men—farmers' sons and mechanics, stimulated by such misplaced eloquence, hold their familiar tools in contempt and make up their mind to become doctors, I emphatically declare that the man who urged them on has done a great wrong to the young men mentioned, to the community, and to the profession. If there is one thing more certain than another it is that this "great and growing country" does *not* want more doctors. I am no hand at statistics, but in Great Britain and Ireland I believe there is a population about as large as ours There there are some twenty thousand doctors, yet from nowhere else is more complaint made of the overcrowded state of the profession. In our country, the last census, if I recollect rightly, made the number of medical men, of all schools, seventy-four thousand—one to every five hundred and forty persons. Considering the gigantic abuses to which medical charities in large cities are subject, and the many paying patients who select long-established physicians of great reputation as their advisers, how miserably minute a fraction of that five hundred and forty comes to each of the juniors.

It cannot be otherwise.

Now I should be the last man in the world to prevent anyone who really has a decided inclination and aptitude for medicine from qualifying himself to practice it; but that inclination ought to be founded on a better basis than the fancy pictures drawn by our professors. They are gentlemen usually—in the cities—in large and remunerative practice, and they must remember that everybody cannot be as they by any amount of work. When every place is full, how are new-comers to be accommodated?

But even if a man know without doubt that medicine is his proper vocation, I warn him, by my example, not to try it if he looks to get his support therefrom. It is true that many a young man has the way made plain for his feet by doting relations and friends, who nurse the second Mott through adversities which would have swamped him unaided. Again, there are instances of blind luck, in which certain individuals stumble right into a certain success; but woe be to him who promises himself a certain leap at success on no other ground than that others have accomplished it before him. He will find the way blocked by scores of eager contestants, each bent on the one object, and which necessarily but one can obtain.

Another strange feature about the profession—a feature which distinguishes it from other learned callings and from trade—is, that money and brains, and time spent in perfecting one's self in medicine, give the man who has thus qualified himself no advantage in competition when seeking the patronage of a community. I was told by physicians of great reputation and first-class metropolitan practice, that by going through Bellevue, and studying awhile abroad, I would put, in a few years, thirty thousand dollars in my pocket. Perhaps that "few years" is not up yet; but certainly in five years the first thousand has not yet come to me. I wish to be distinctly understood as putting the incalculable and no-where-else-to-be-found advantages of hospital interne services on a higher level than that of a mere monetary investment, nor did I ever suppose that study at home or abroad would ever enable me to secure anything like the returns which flow into the coffers of a certain carpenter, turned quack, of whom I have knowledge. Nevertheless, there is certainly some excuse for the dissatisfaction of a man who, on the earnest recommendation of several veteran Æsculapians, reduced still farther his slender remaining means, that he might secure a thorough training for his work, and in five years after finds that the army of medical college graduates, whose whole education comprises the two courses of lectures, and reading with a preceptor (?), will seldom be behind, and in many instances is ahead of him. A well-meaning gentleman, who, until three years ago, handled the plow with adept hand, and, as he himself informed me, with great seeming satisfaction, "cudn't neither read ner write at thet time," has just put up his name, with the cabalistic M. D. after it, on the house next to mine. He showed me a diploma, certifying that J. M. had been granted the degree of doctor in medicine for numerous sufficient reasons, by the Faculty of the Medical Department of the University of Round-Holed Corners. The diploma was in Latin, such as Terence, or any *late* Latin author would have rejoiced to produce. J. M. confessed to me, under his breath, that his early education having been neglected—rather superfluous information—he had never been able to learn just how much he did know. This I interpreted as a modest request to have the document translated. That I did; and though neither English or American was his language, but the beautiful pastoral dialect spoken in the Far-Down East, he seemed to understand. Thenceforth, whenever I passed his window, he was seen wrapped in ecstasy, gazing intently at the diploma as at an object of devotion, with a Latin dictionary which I had lent him in his hand. At last he mastered the words for himself, and rushing in to me, with the elegantly framed parchment in his hand, he pointed to it in triumph and said:

"Wall neow, I know all about it, an' I'm a doctor neow, jes like yeow; ain't I?"

This man is beating me all to pieces in practice.

"We wos both docteers," said a farmer, "an' won docteer don't kneow more'n another."

I am aware that many will impute the causes of my non-success to myself, and will have it so, say what I may. But I cannot subscribe to their opinion, savor as my opposition will of self-sufficiency. The best proof that I am right is found in my knowledge of the career of no less than ten first-rate men, who likewise went through hospitals. I have been at the pains to consult these gentlemen, and, though my lucky brethren may find in the substantial agreement of the unsuccessful ones' views a point for joking about misery loving company and foxes losing tails, I beg to suggest that these views are the product of a very bitter experience, and at least worthy of attention.

I would like to ask those favored gentlemen who lecture on the needs of this great and growing country for more doctors, if they were ever at a place within its limits where there was a deficiency of the article? "A deficiency of the good article." Ah! very well; but is it not just this wholesale making of doctors by the medical schools and their professors which brings about this state of things? What would you have? Surely it is the interest of the schools to have as many students as possible, and the interest of each professor to graduate as many of his class as he can. One of the colleges in New York turned out a man as M. D. who, to my personal knowledge, could not tell the branches of the aortic arch, and whose examination in other departments than anatomy was proportionally brilliant. It is time some one should speak out, and as one who knows, I arraign our medical colleges as opening the ranks of the profession to a body of men who, as a class, are absurdly incapable, and, in consideration of the interests of life and death to be committed to their care, criminally incapable. I have known of the leading medical college of New York, some years since, graduating a man who never soiled his hands in the dissecting-room, never touched a body, and whose reading comprised only that labor-saving machine, Neill and Smith's Compendium; that is he was never known to have any other medical work, and if he did, it was certain that N. and S. taught him all he knew. This man got a certificate of study from a doctor in whose office the student never entered, and systematically "cut" lectures. Of course this bogus doctor, as bogus as the possessor of any bought diploma, this "*virum probum*," had a brief career. Not at all. The time that other and more conscientious men gave to study, he gave to developing the qualities vulgularly known as "brass" and "cheek;" to influencing politicians successfully in his favor, and to drawing up "*Rules to be Observed during Treatment;*" and to-day his income is not less than ten thousand dollars yearly. Can we wonder that the community favors quacks? Nay, I have no hesitation in saying, and I say it boldly, that I would prefer at any time the services of an intelligent "quack" to such a "regular" doctor. I know that parallel cases exist in all the institutions in the great cities, and if this be the case there, what must be the depth of knowledge of the country school graduate, who is from a free school, from where the "professors" go out in the highways and hedges and compel students to come in?

The sublime trust with which some members of the profession speak of the general acceptance of a medical college degree as an educational guarantee is a patent instance of faith without works, What nonsense the professor utters, who says to the President, in the face of an admiring audience, smiling "graduates," and after music by the band, "These candidates have shown by their examinations," etc. A large proportion have shown great ignorance by their examinations; have copied stale text-books to construct that wonderful literary production, the "Thesis;" have given certificates of three years' study and of a moral character, from one whom they may have seen once in their lives, and have attended mythical lectures. How does the faculty know that candidates have attended two courses of lectures? The faculty is sure of one thing, and you may be sure of it. The candidates have shown, by handing over greenbacks, that they have *paid* for the lectures, which they may have never heard; for dissection, which they may have never prosecuted; and for graduation, which they are sure to get. I know this picture of a first-class medical college is not the received one. I only know it to be true. Why! you are requiring Spartan virtue of men when you expect them to stickle about how much a man knows, when he has paid for what he expects to get, and when "putting him through" is the condition of getting to more pay for similar easy graduation. There is no doubt of it. Our medical colleges are run with one great object in view—to make money for the professors, and the competition between them is fierce, not as to the quality, but the quantity of the material they turn out.

When I mention these facts to the nabobs of the profession, I do not find them denied, but it is usually said, "Oh! yes; but the inferior men drop off, and if a man really sets about to learn something for himself, he must distance the ordinary run of new graduates in a short time." For the reasons gone over I believe this view to be all fiction. It is useless to expect the laity to inquire whether a man has had hospital experience, and has studied abroad. If the absurd "ethics" of the profession permitted a man to tell people his qualifications in public print, it would be different. This exclusion from advertising I deem sufficient answer to those who contend that medicine is no worse than any other business or profession, law perhaps excepted. What earthly objection is there to advertising? "Honor, dignity of the profession," says the great and wealthy Dr. Blank. Of course, Dr. Blank, you don't need it; but how about us poor devils? What reason is there for my not letting people know I have had special opportunity to study eye diseases, or fractures, or what not? We stand by, and let charlatans reap all the benefit of this great means of success. But why let medical colleges advertise? DIPLOMA.
—*Medical Record.*

